# Supplementary figures and images for: Targeting AAV vectors to the central nervous system by engineering capsid–receptor interactions that enable crossing of the blood–brain barrier
Source: PLoS Biol. 2023 Jul 19;21(7):e3002112. doi: 10.1371/journal.pbio.3002112 (PMC10355383; doi:10.1371/journal.pbio.3002112)

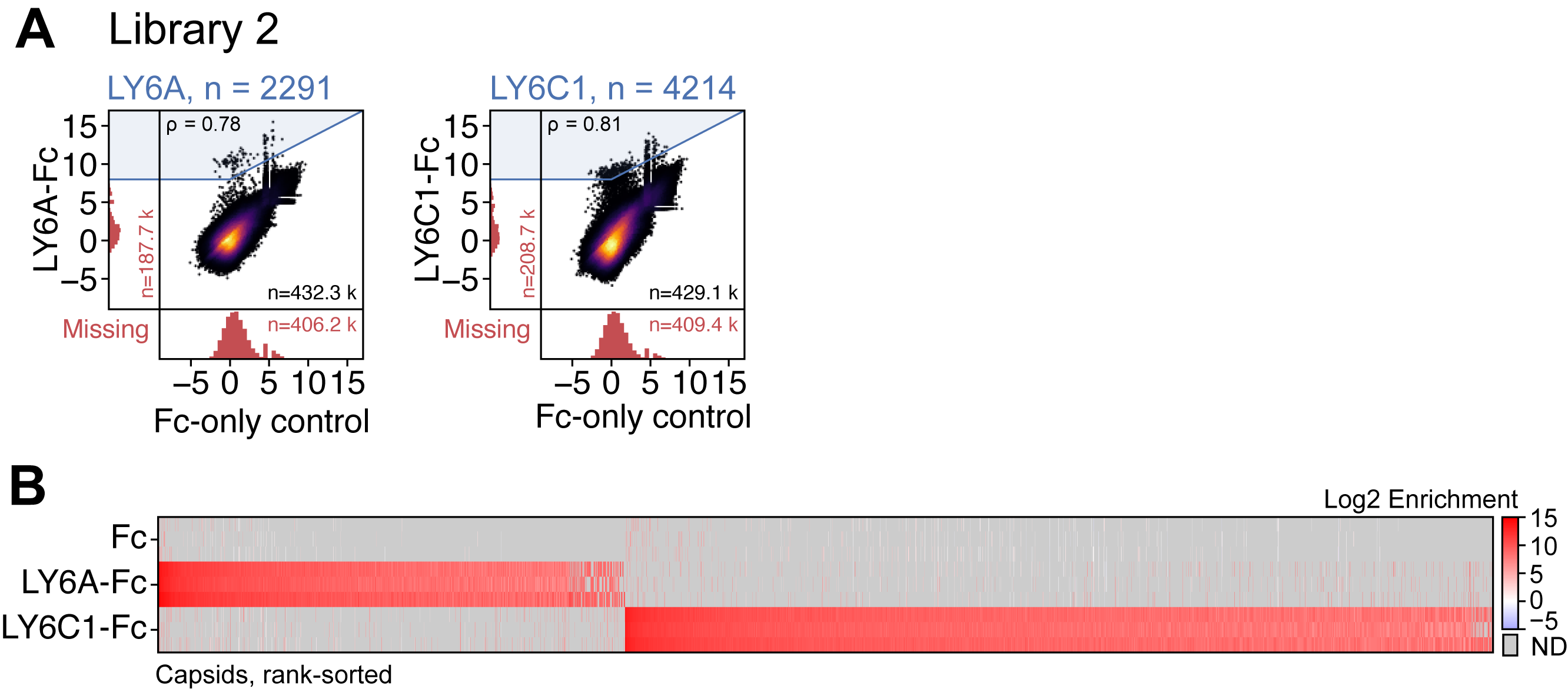

Supplement: S1 Fig — (A) The variant log2 enrichment (average RPM between replicates, normalized to the starting library RPM) plotted between LY6A-Fc or LY6C1-Fc versus the Fc-only control. The capsids detected in both assays are displayed in the upper-right quadrant. Missing variants from either assay are displayed in the marginal quadrants. (B) The log2 enrichment of selected variants highlighted in blue in (A) with each replicate’s enrichment plotted in separate rows (n = 3). ND = not detected. The underlying data supporting S1 Fig can be found at https://doi.org/10.5281/zenodo.7689794: library2_pulldown.csv. (TIF) [file pbio.3002112.s001.tif]

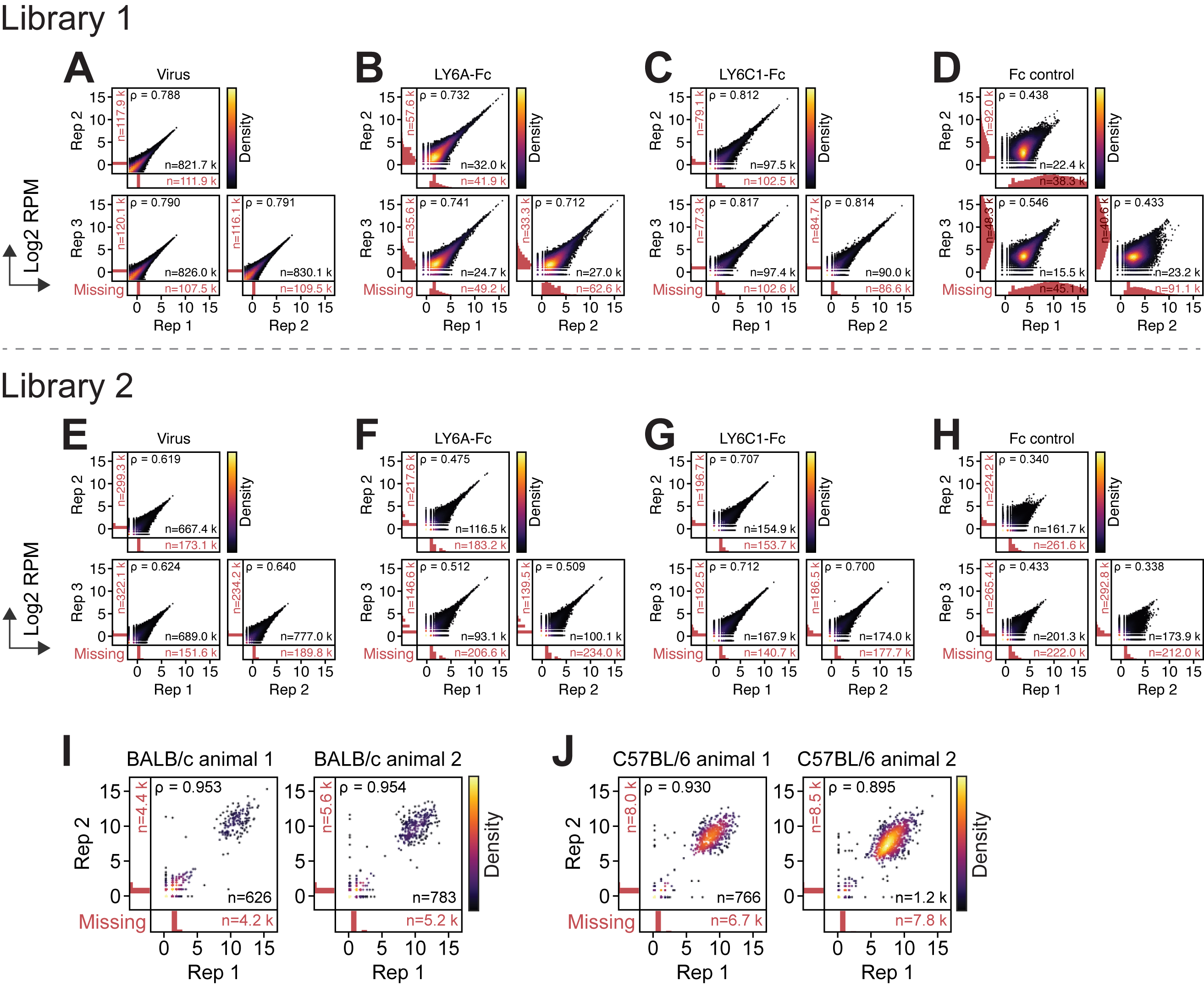

Supplement: S2 Fig — (A–D) Screen of Library 1 replicability of the log2 RPM of the (A) starting virus library, (B) LY6A-Fc, (C) LY6C1-Fc, and (D) Fc-only control. (E–H) Screen of Library 2 replicability of the (E) starting virus library, (F) LY6A-Fc, (G) LY6C1-Fc, and (H) Fc-only control. (I, J) Replicability of separate RNA extractions (n = 2 extractions per mouse strain) within each mouse strain (n = 2 mice) for (I) BALB/cJ and (J) C57BL/6J. The capsids detected in both replicates are displayed in the upper-right quadrant. The missing variants from either replicate are displayed in the marginal quadrants. The underlying data supporting S2A–S2D Fig can be found at https://doi.org/10.5281/zenodo.7689794: library1.csv; S2E–S2H Fig at https://doi.org/10.5281/zenodo.7689794: library2_pulldown.csv. (TIF) [file pbio.3002112.s002.tif]

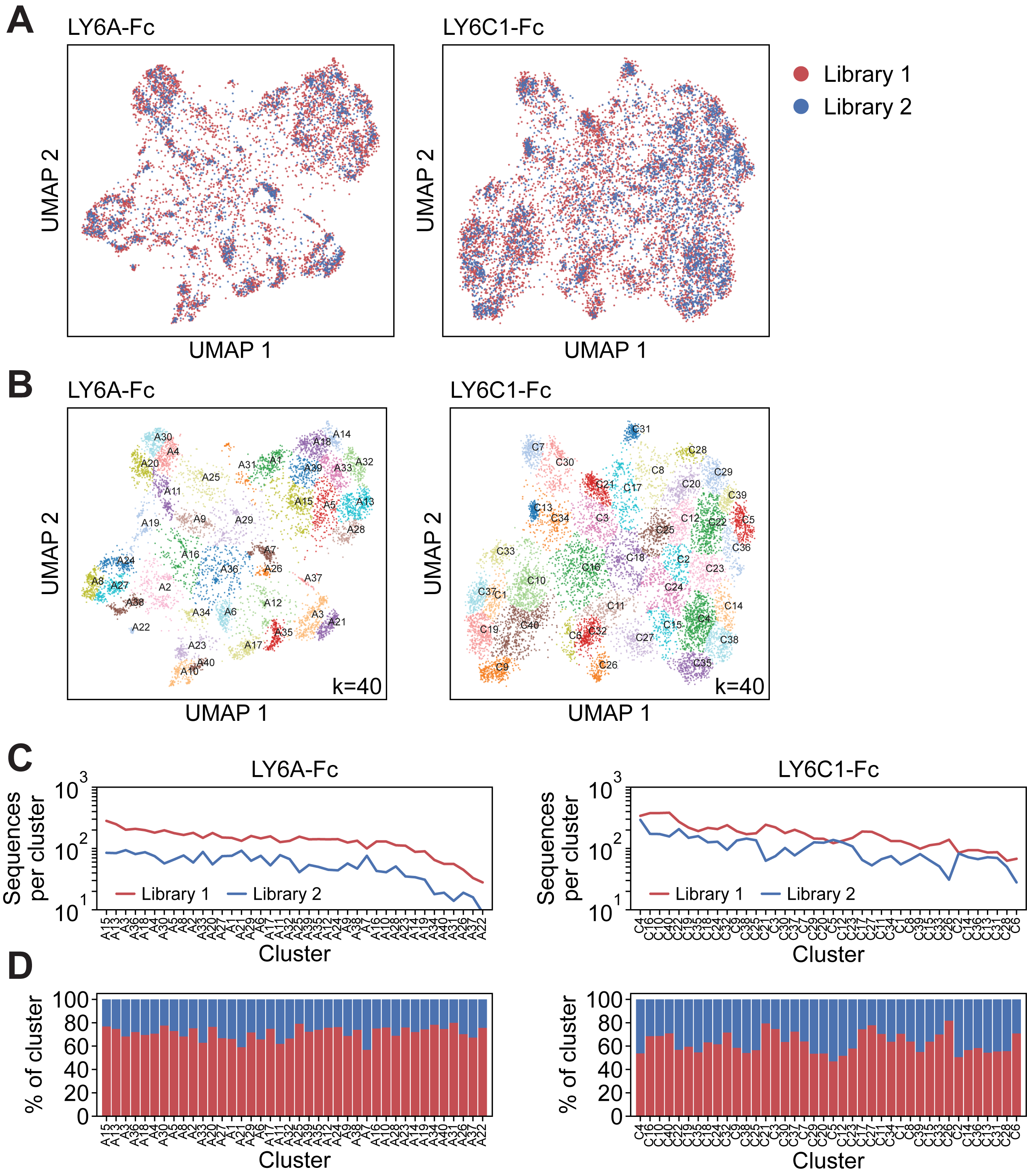

Supplement: S3 Fig — (A) The joint UMAP embedding of target-specific 7-mer sequences with sequences colored according to experiment. (B) The clustering (Gaussian mixture model, k = 40) on the joint embedding. (C, D) The number (C) and percentage (D) of 7-mer sequences by the Round 1 pull-down screen from each library, per cluster (sorted from left to right by the number of sequences per cluster). The underlying data supporting S3 Fig can be found at https://doi.org/10.5281/zenodo.7689794: LY6A_joint_umap_l1_l2.csv and at https://doi.org/10.5281/zenodo.7689794: LY6C1_joint_umap_l1_l2.csv. (TIF) [file pbio.3002112.s003.tif]

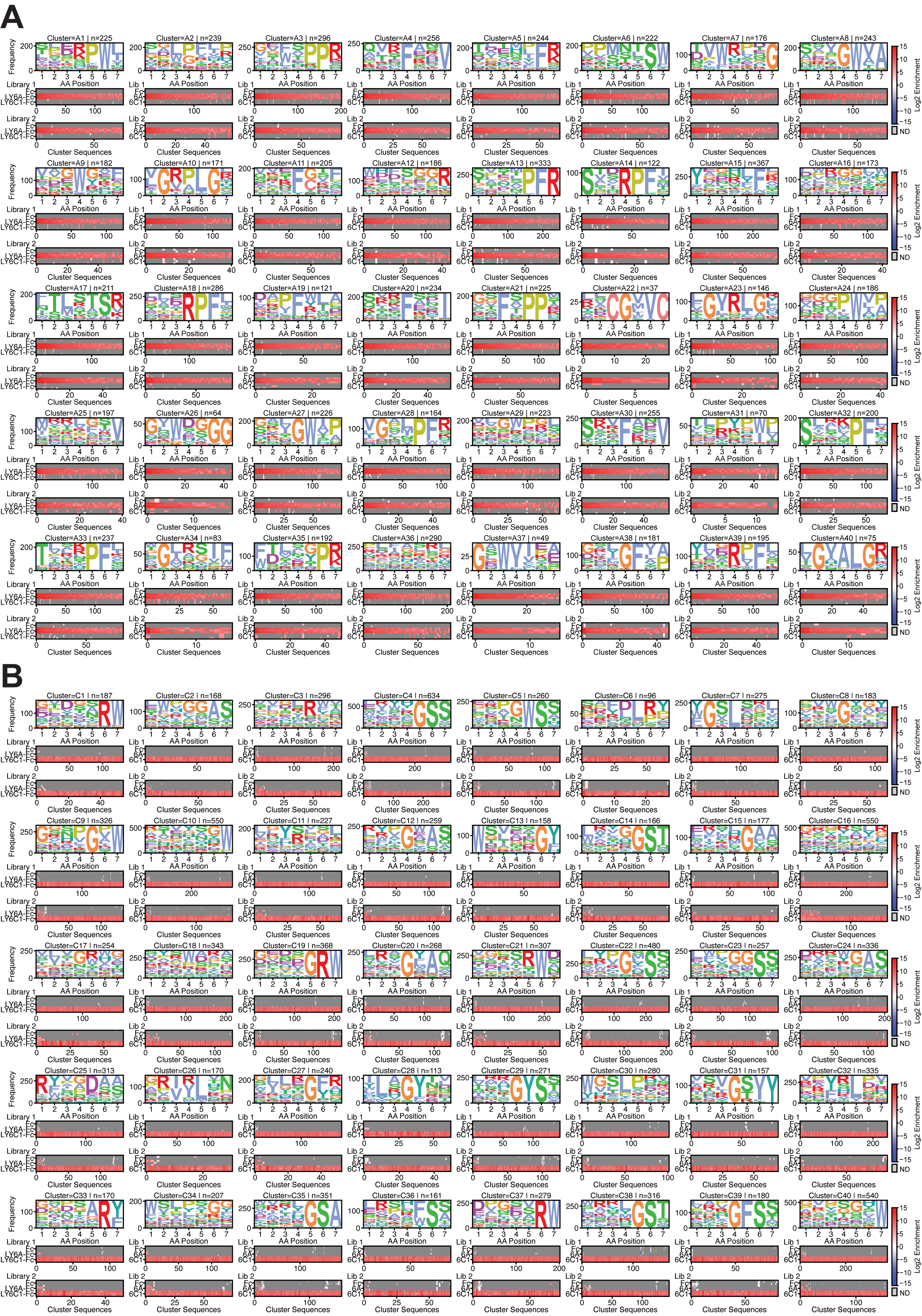

Supplement: S4 Fig — (A) LY6A or (B) LY6C1 cluster sequence logos and the corresponding heatmap of log2 enrichments for sequences in each cluster for the Fc-only control, LY6A-Fc, and LY6C1-Fc. The underlying data supporting S4 Fig can be found at https://doi.org/10.5281/zenodo.7689794: LY6A_joint_umap_l1_l2.csv and at https://doi.org/10.5281/zenodo.7689794: LY6C1_joint_umap_l1_l2.csv. (TIF) [file pbio.3002112.s004.tif]

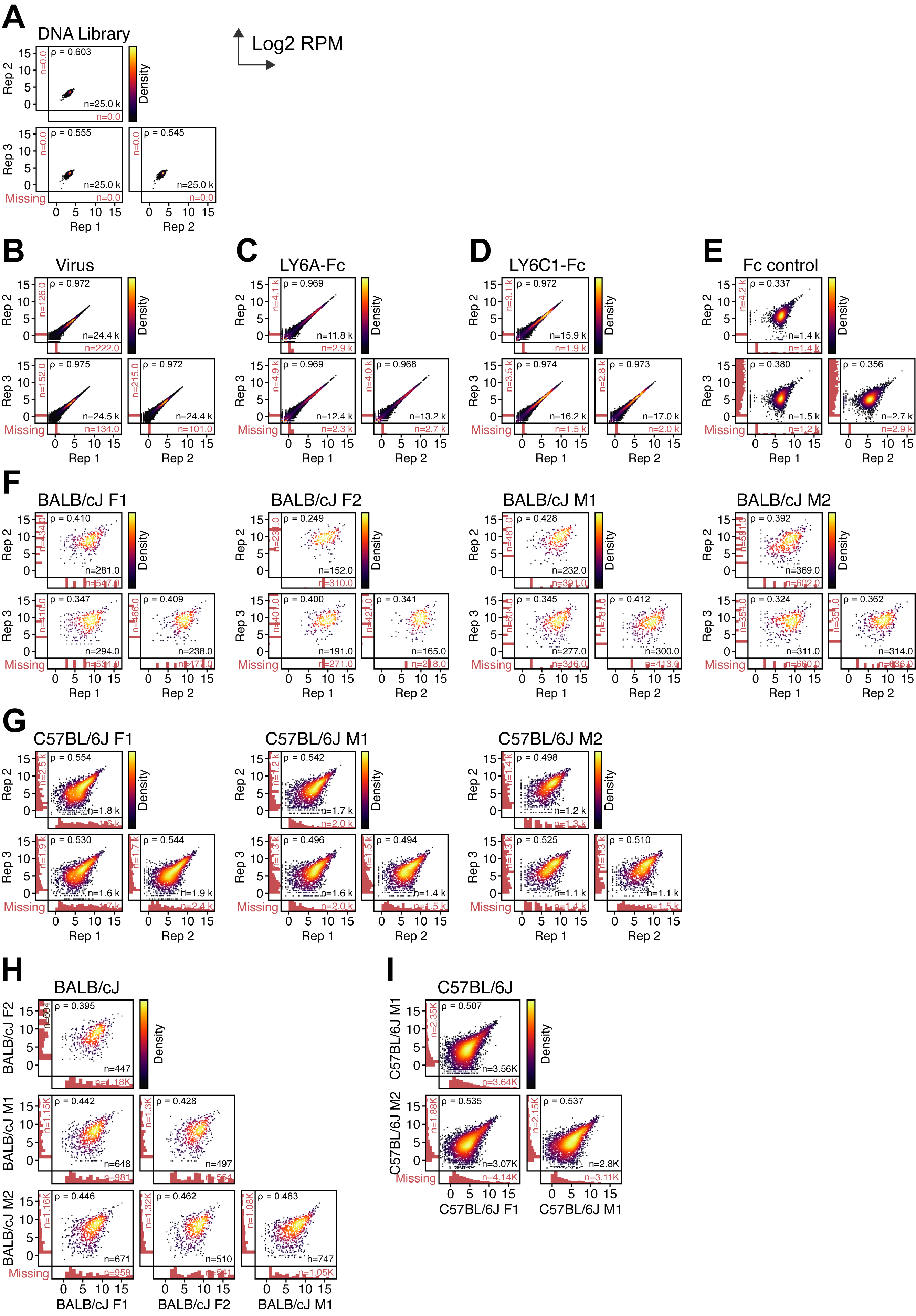

Supplement: S5 Fig — The plots show the replicability of the log2 reads per million (RPM) of the (A) DNA (plasmid) library, (B) virus library, (C) LY6A-Fc, (D) LY6C1-Fc, and (E) Fc-only control. The capsids detected in both replicates are displayed in the upper-right quadrant. The missing variants from either replicate are displayed in the marginal quadrants. The replicability of separate RNA extractions are shown for (F) BALB/cJ (4 mice [F1, F2, M1, M2], n = 3 extraction replicates per animal) and (G) C57BL/6J (3 mice [F1, M1, M2], n = 3 extraction replicates per animal). The mean RPM from the extraction replicates between animals were compared for (H) BALB/cJ and (I) C57BL/6J. The underlying data supporting S5 Fig can be found at https://doi.org/10.5281/zenodo.7689794: round2_codons_merged.csv. (TIF) [file pbio.3002112.s005.tif]

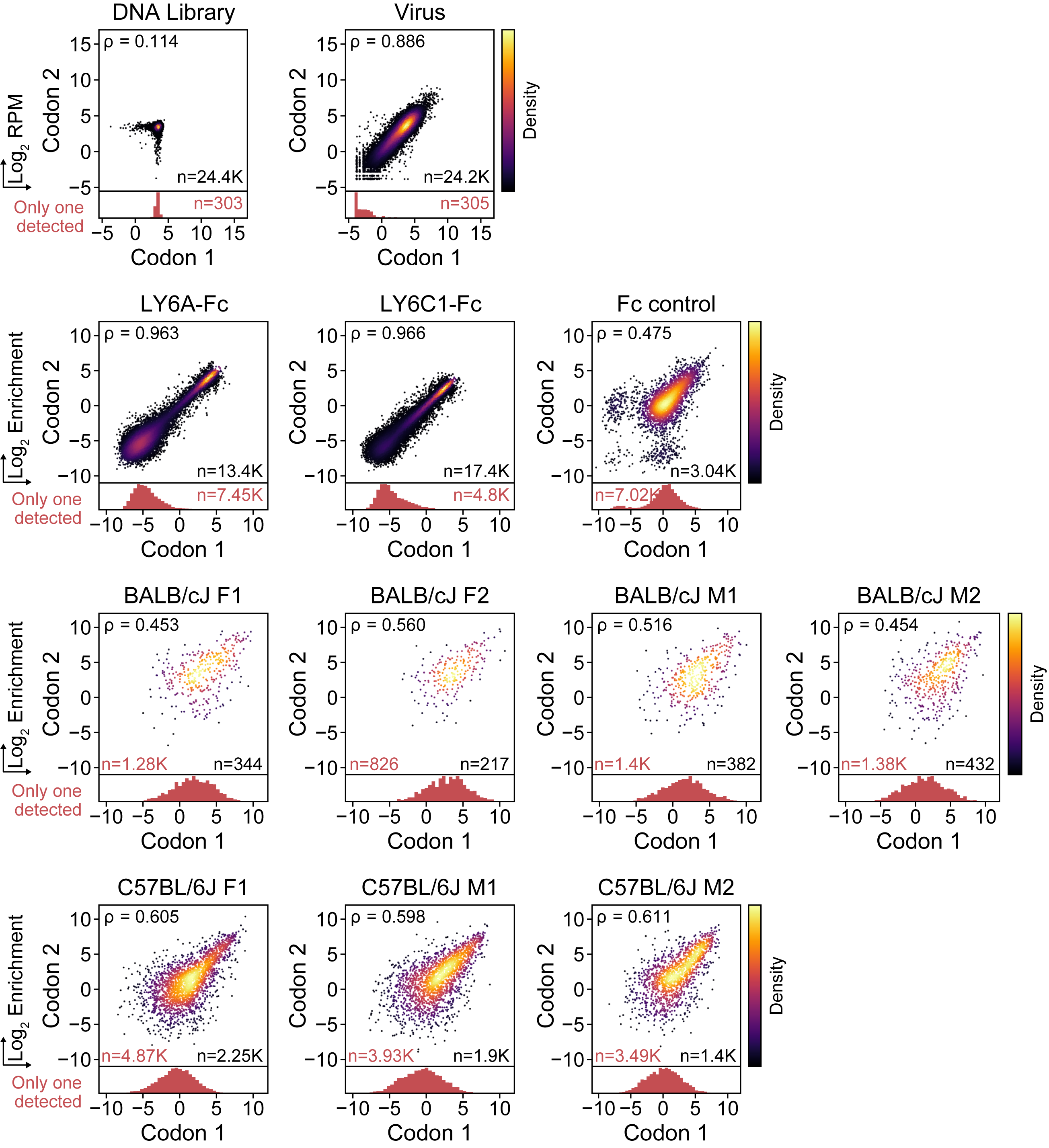

Supplement: S6 Fig — The values shown are log2 RPM for the DNA library and virus library samples, and log2 enrichment for the in vitro and in vivo samples. Sequences within pairs of 7-mer AA replicates (codon 1 and codon 2) were randomly assigned to either the x- or the y-axis, with the exception of AA sequences missing their partner within a replicate pair that are assigned to the x-axis and plotted in the histogram below each plot. The underlying data supporting S6 Fig can be found at https://doi.org/10.5281/zenodo.7689794: round2_codons_separate.csv. (TIF) [file pbio.3002112.s006.tif]

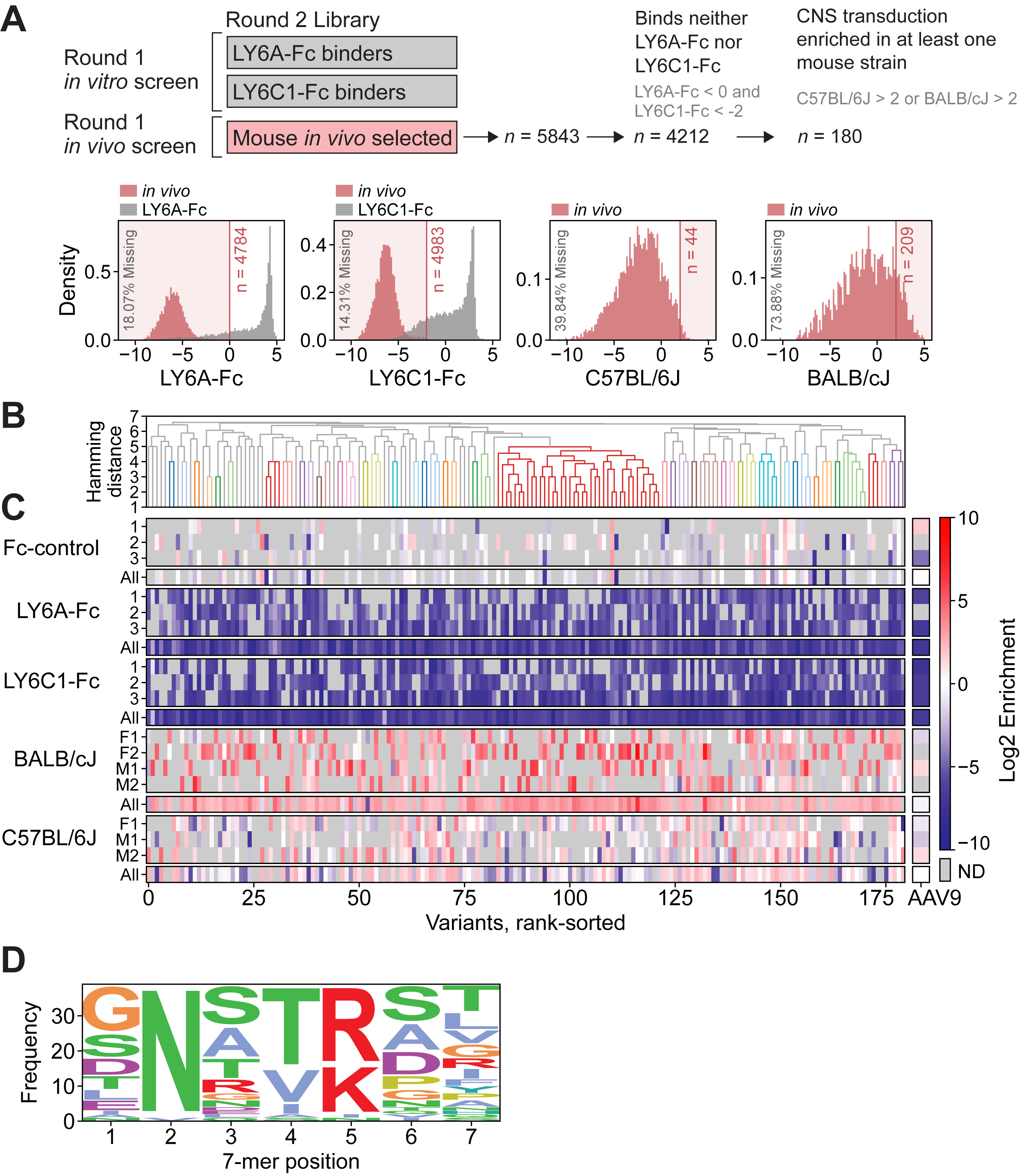

Supplement: S7 Fig — (A) Round 2 variants identified in the Round 1 in vivo screen (red) were filtered by the thresholds shown for low binding to LY6A-Fc (LY6A-Fc binders are shown in gray), low binding to LY6C1-Fc (LY6C1-Fc binders are shown in gray), and high CNS transduction in either C57BL/6J or BALB/cJ mice. This combined filtering yielded 180 variants. (B) Hierarchical clustering of the 180 variants by hamming distance (linkage = average, cutoff = 5) yielded 1 large cluster (red, center, n = 39). (C) Log2 enrichment is shown for each variant ordered by the clustering tree in (B) for in vitro binding of the Fc-only control, LY6A-Fc, LY6C1-Fc, and CNS transduction in BALB/cJ or C57BL/6J mice. (D) The sequence motif of the center red cluster (n = 39) in (B) shows a clear pattern of *N*[T/V/I][R/K]**. The underlying data supporting S7 Fig can be found at https://doi.org/10.5281/zenodo.7689794: round2_codons_merged.csv. (TIF) [file pbio.3002112.s007.tif]

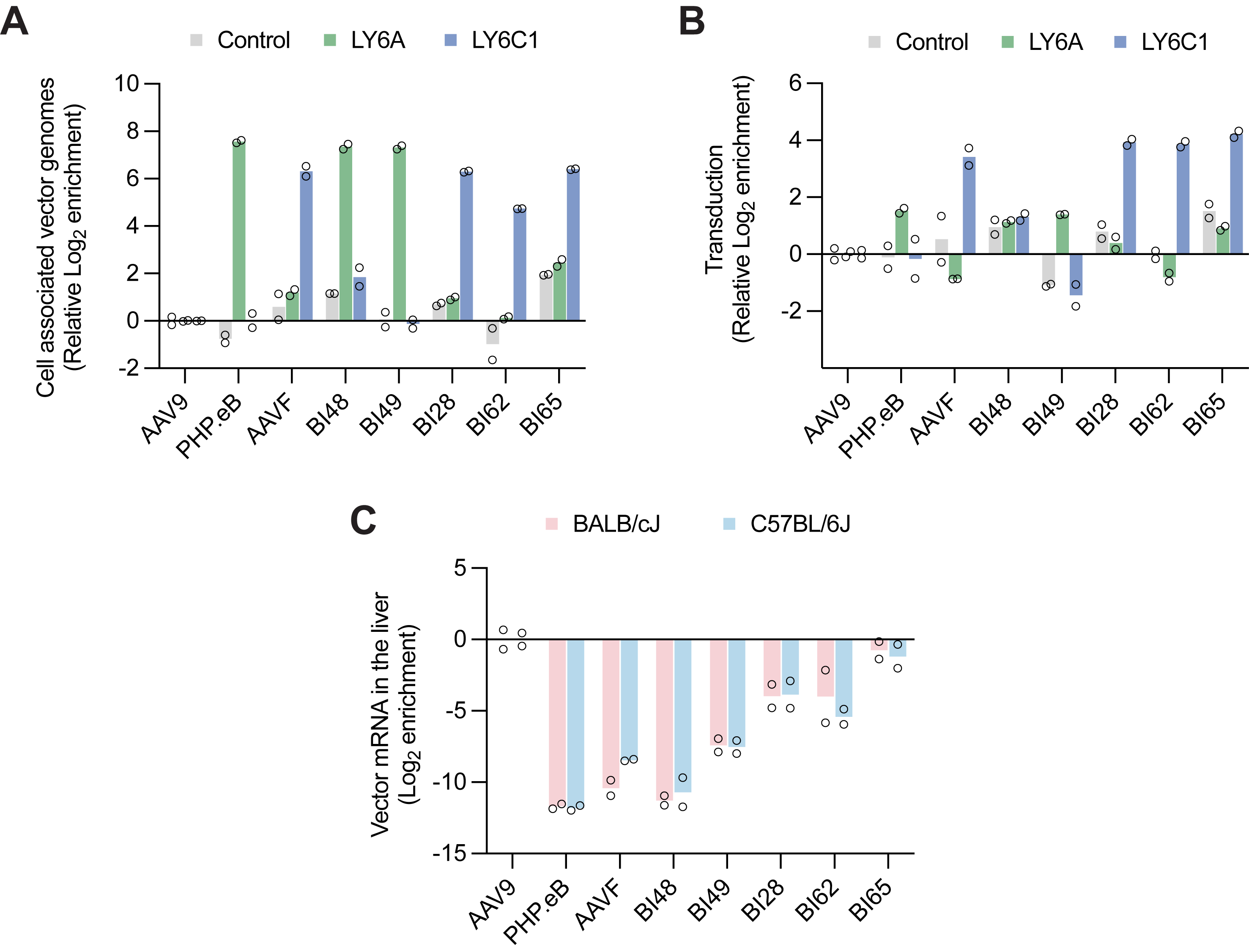

Supplement: S8 Fig — Binding (A) and transduction (B) of HEK293 cells expressing Ly6a, Ly6c1, or a control (GFP) cDNA by the indicated AAVs. (A, B) The enrichment of the reference capsids (AAV9, AAV-PHP.eB, or AAVF) in comparison with the capsids identified in this study (BI48, BI49, BI28, BI62, BI65) observed in a pooled library study are shown. (C) Liver transduction by the indicated variants from the same library tested in BALB/cJ and C57BL/6J mice is shown. Each graph shows the mean enrichment (bars) of each capsid normalized to AAV9, with individual values from 7-mer AA replicates (encoded by different nucleotide sequences) shown as individual data points (circles) (n = 4 animals/per group). The underlying data supporting S8A Fig can be found at https://doi.org/10.5281/zenodo.7689794: S8_A_HEK_binding.csv; S8B Fig at https://doi.org/10.5281/zenodo.7689794: S8_B_HEK_transduction.csv; S8C Fig at https://doi.org/10.5281/zenodo.7689794: S8_C_liver_transduction.csv. (TIF) [file pbio.3002112.s008.tif]

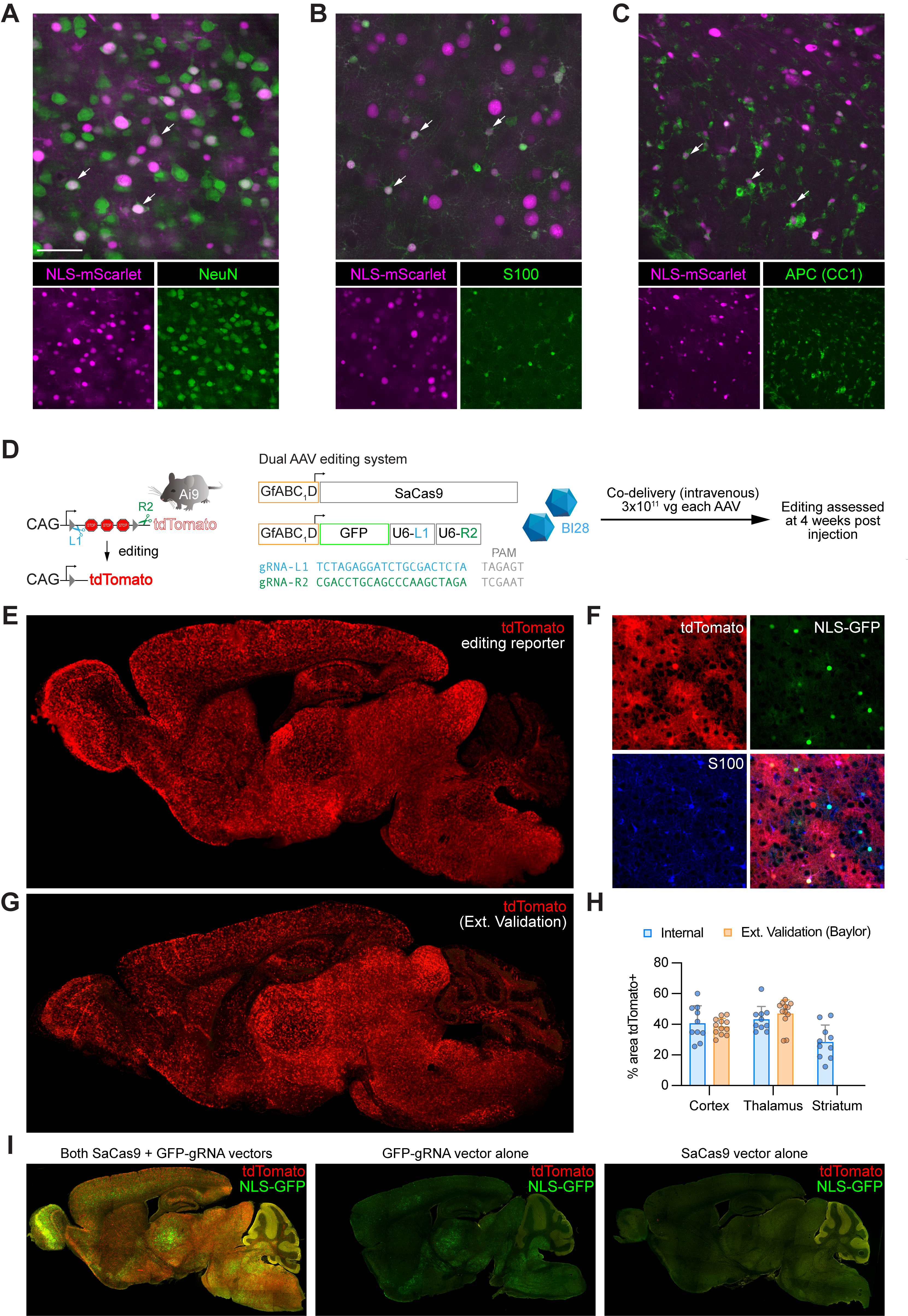

Supplement: S9 Fig — (A–C) Immunofluorescence analysis of transduced cell types 4 weeks after adult intravenous administration of 1 × 10^11^ vg/animal AAV-BI28:CAG-NLS-mScarlet-2A-Luciferase-WPRE-pA. Representative images show colocalization of mScarlet+ cells (magenta) with NeuN (A, green) and S100 (B, green) in the cerebral cortex and CC1+ cells (C, green) in the corpus callosum. Arrows highlight example transduced marker+ cells. Scale bar is 50 μm. (D) The schematic shows the dual AAV gene editing system designed to remove the stop cassette and turn on tdTomato expression in Ai9 reporter knock-in mice. The first rAAV expresses SaCas9 from the astrocyte-selective GfABC1D promoter. The second rAAV expresses GFP from the same promoter and 2 tandem U6-driven gRNAs (L1 and R2, with the indicated spacer and PAM sequences). Both rAAV genomes were packaged into AAV-BI28 and co-administered to Ai9 tdTomato reporter mice at 3 × 10^11^ vg/mouse (total dose 6 × 10^11^ vg/mouse) and editing was assessed 4 weeks later. (E) Representative whole sagittal brain section images (top: Deverman laboratory results, bottom: BCM-Rice SATC external validation) show Ai9 locus editing as assessed by tdTomato native fluorescence. (F) Colocalization of tdTomato expression with GFP (transduction marker) and S100 in cortical astrocytes are shown. (G) Independent validation of gene editing with the AAV-BI28 vectors in Ai9 mice by researchers at the BCM-Rice SATC in coordination with the NIH Somatic Cell Genome Editing Consortium. (H) The graph shows the quantification of CNS astrocyte editing measured by the fraction of tdTomato+ area above threshold within the indicated brain regions (mean ± SD, Internal cohort, n = 6 females and n = 4 males; Baylor validation cohort, n = 6 females and n = 6 males). (I) Whole sagittal brain sections show that administration of both vectors is required to achieve gene editing. Note, the weak signal present in both red and green channels that is most notable in the cerebellum in the SaCas [file pbio.3002112.s009.tif]

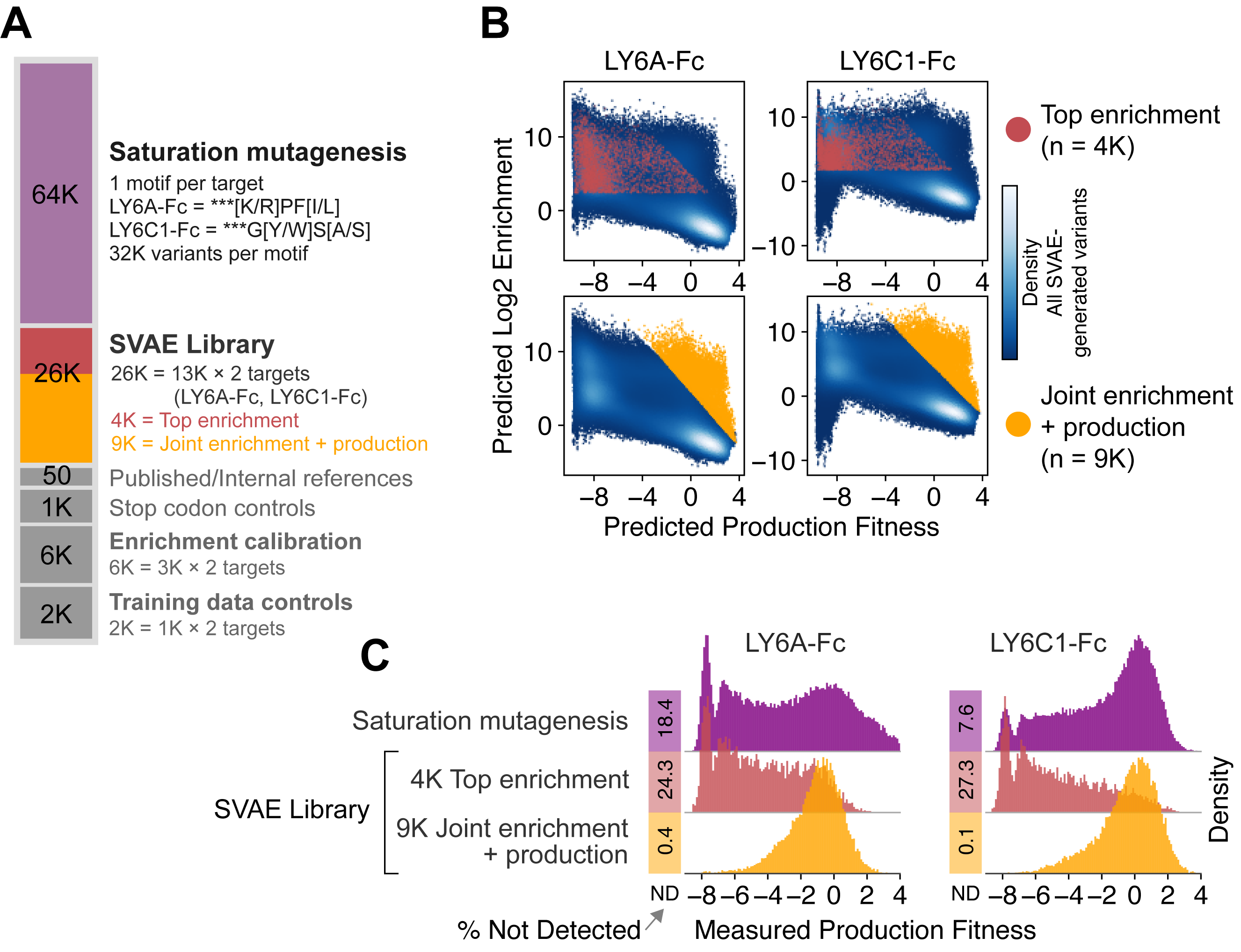

Supplement: S10 Fig — (A) The combined SVAE and saturation mutagenesis library is composed of saturation mutagenesis variants generated from 1 motif per target (LY6A ***[K/R]PF[I/L], LY6C1 ***G[W/Y]S[A/S]) with 32K variants per motif; 13K SVAE-generated variants per target; 50 previously characterized variants from our group and the literature; 1K variants with stop codons to assess cross-packaging; 6K variants (3K per target) that were evenly selected across low-to-high enrichment bins to calibrate the enrichment scores from this library to the library used to train the SVAE models (Round 1, Library 1); 2K variants (1K for each target) that were randomly chosen from the SVAE training data (i.e., variants with non-zero RPM from Round 1) as training data controls. (B) The predicted binding enrichment and predicted production fitness for SVAE-generated variants (150K generated in silico per target) are shown. Included in the SVAE Library were the 4K variants with the top predicted binding enrichment according to the SVAE (red), as well as the top 9K variants according to a joint score of predicted binding enrichment and predicted production fitness (yellow). (C) The virus library shown in (A) was produced and the distributions of the measured production fitness of the saturation mutagenesis-generated and SVAE-generated variants are shown. The underlying data supporting S10B Fig can be found at https://doi.org/10.5281/zenodo.7689794: LY6A_SVAE_generated_sequences.csv and 10.5281/zenodo.7689794: LY6C1_SVAE_generated_sequences.csv; S10C Fig at https://doi.org/10.5281/zenodo.7689794: SVAE_SM_library_codons_separate.csv. (TIF) [file pbio.3002112.s010.tif]

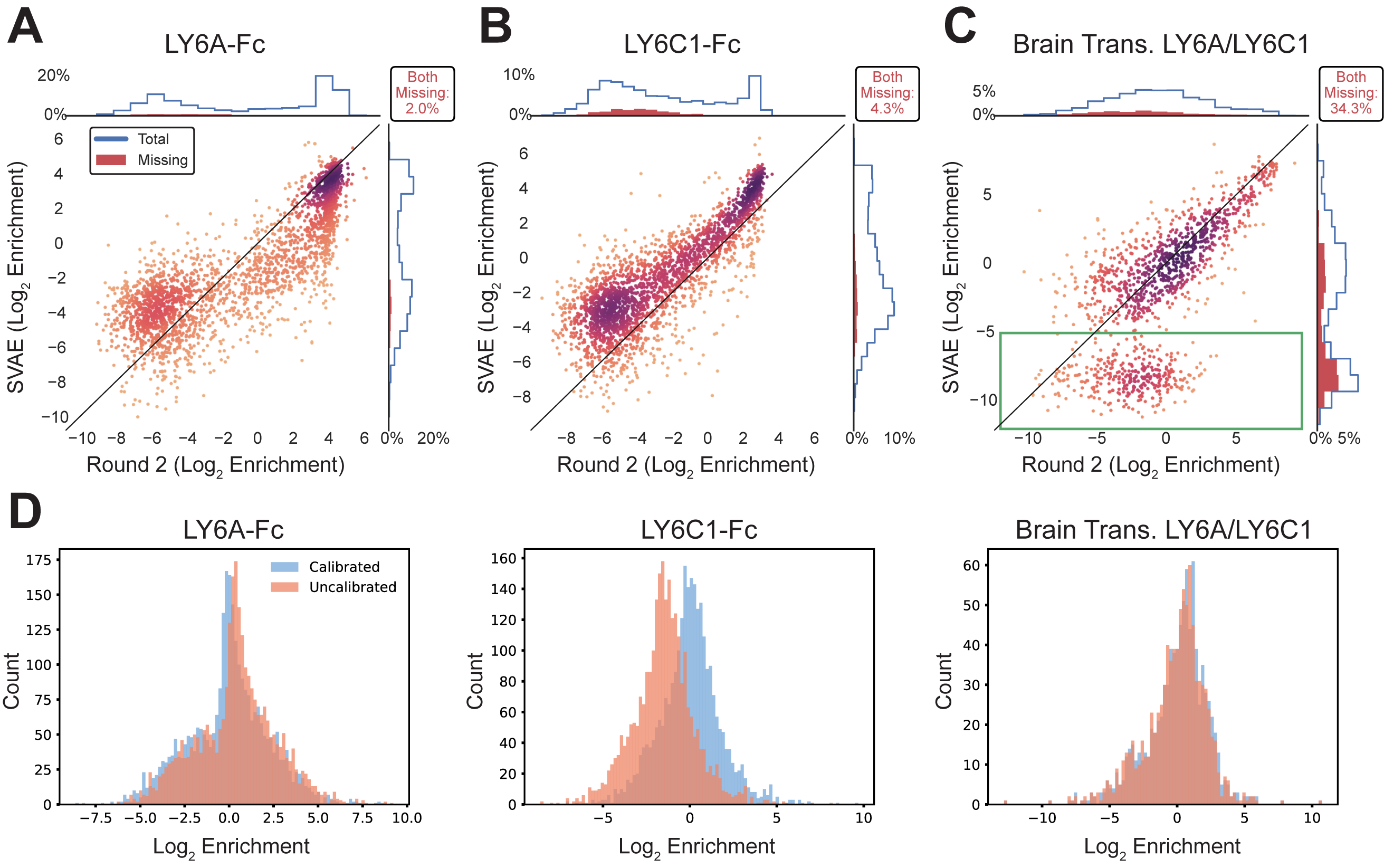

Supplement: S11 Fig — Library variant enrichment scores are relative because they are derived from comparisons to the other members of the same library. The scores of variants from separate libraries were calibrated by computing a single value that adjusts these scatter plots (A–C) on the y-axis to minimize error (see Materials and methods, S12–S23 Data). (A, B) Plots of the enrichment scores within the SVAE library versus the Round 2 library for the uncalibrated LY6A- and LY6C1-binding sequences that are common to both libraries. Histograms on the top and right margins show the distribution of total variants (blue) and variants missing in one of the assays (red). (C) The same as in (A, B), but for the brain transduction assay. For the brain transduction assay, both libraries contain LY6A- and LY6C1-binding variants so a single calibration value was applied. Points in the green box were dropped when computing the calibration. We hypothesize that this discrepancy arose from the Round 2 library being sequenced more deeply. (D) Histograms of pre- and post-calibration enrichment for each assay. Calibration values are as follows: LY6A: −0.37, LY6C1: 1.50, brain transduction, combined LY6A/LY6C1: 0.14. The amount of shift between the pre- and post-calibration histograms corresponds to the calibration value for each assay. The underlying data supporting S11 Fig can be found at https://doi.org/10.5281/zenodo.7689794: round2_codons_merged.csv and 10.5281/zenodo.7689794: SVAE_SM_library_codons_merged.csv. (TIF) [file pbio.3002112.s011.tif]

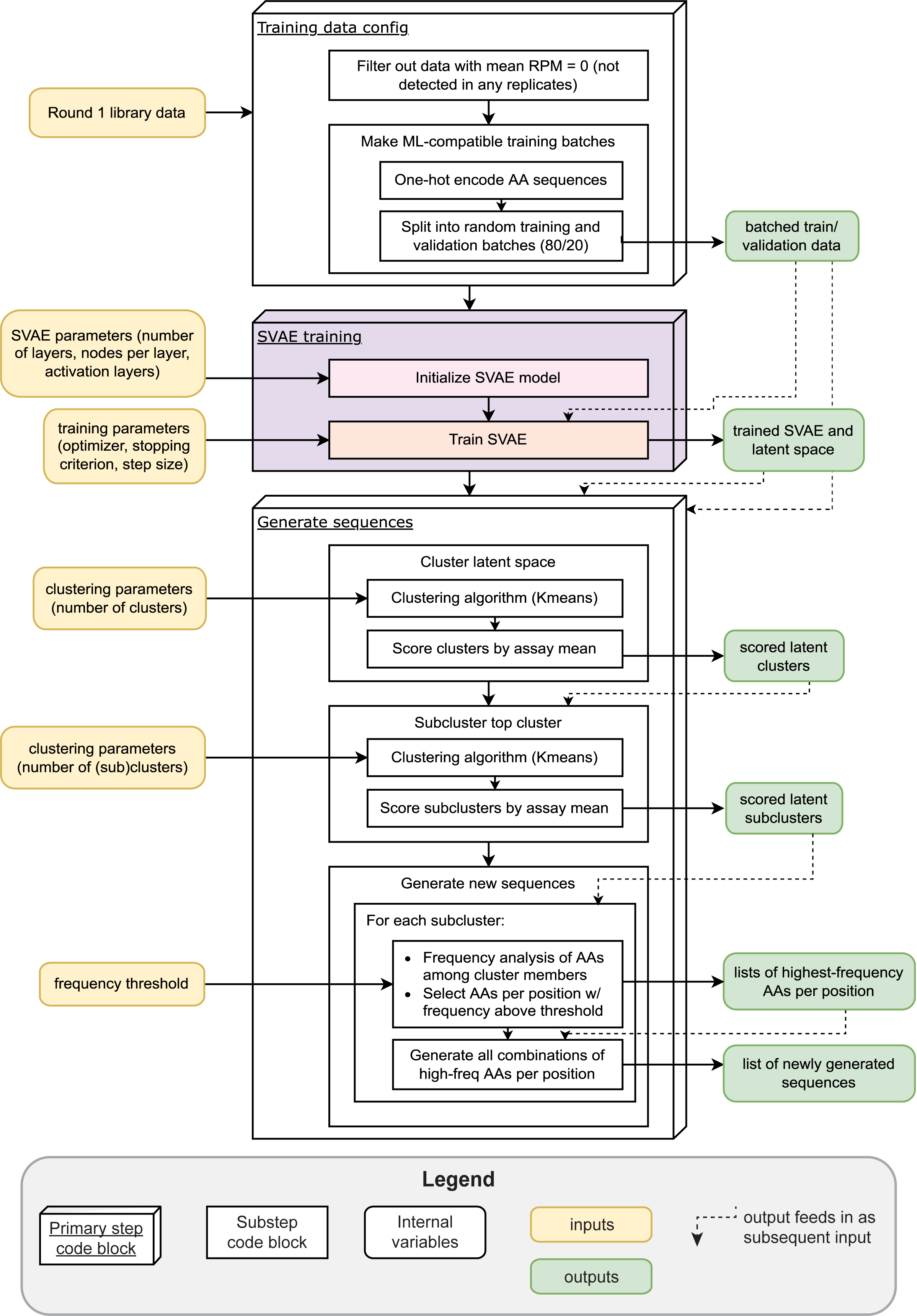

Supplement: S12 Fig — (A) A schematic of the complete SVAE-based sequence generation procedure, including (1) the processing of training data, (2) SVAE training, and (3) sequence generation using the SVAE latent space. (TIF) [file pbio.3002112.s012.tif]

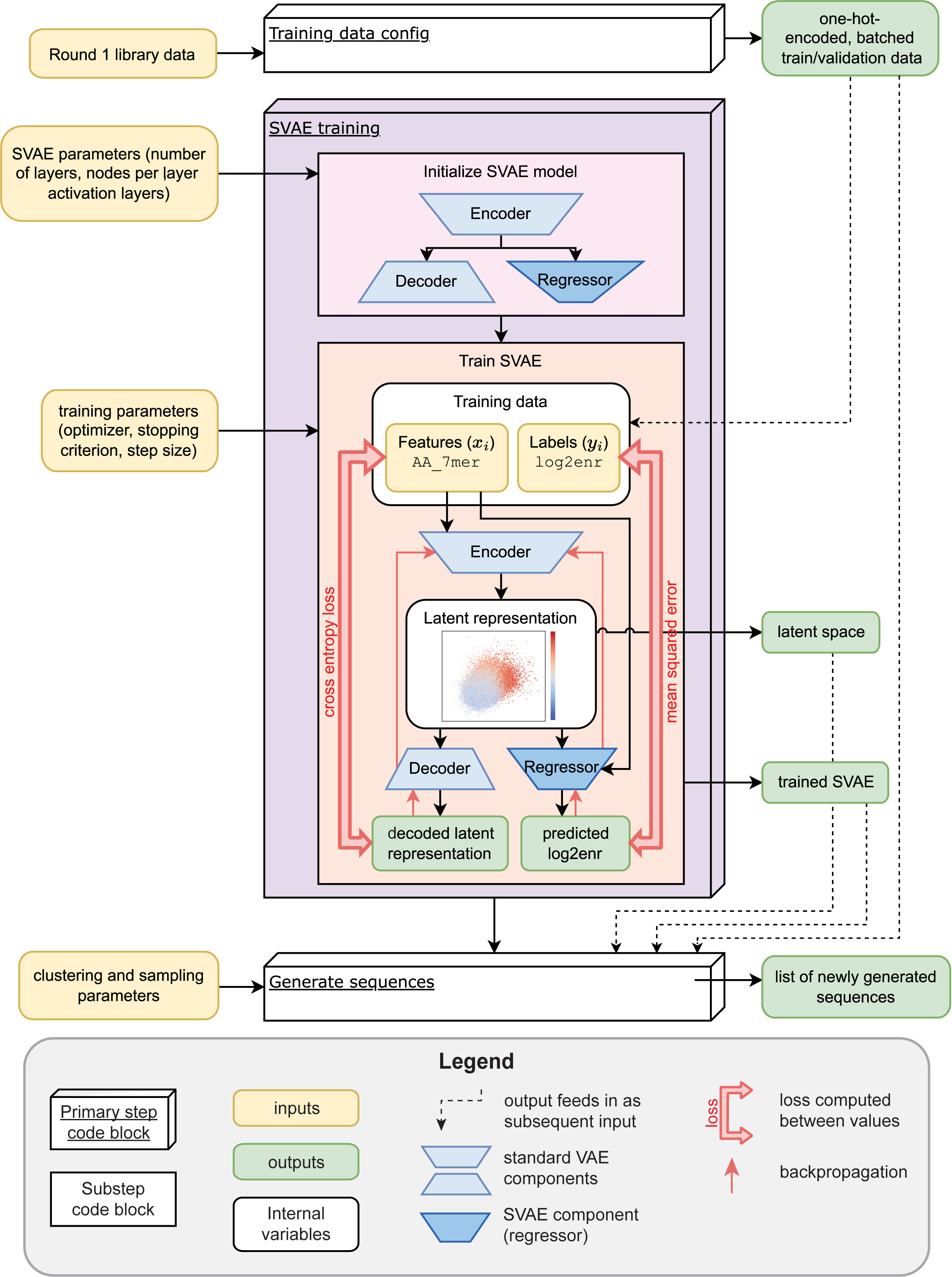

Supplement: S13 Fig — (TIF) [file pbio.3002112.s013.tif]

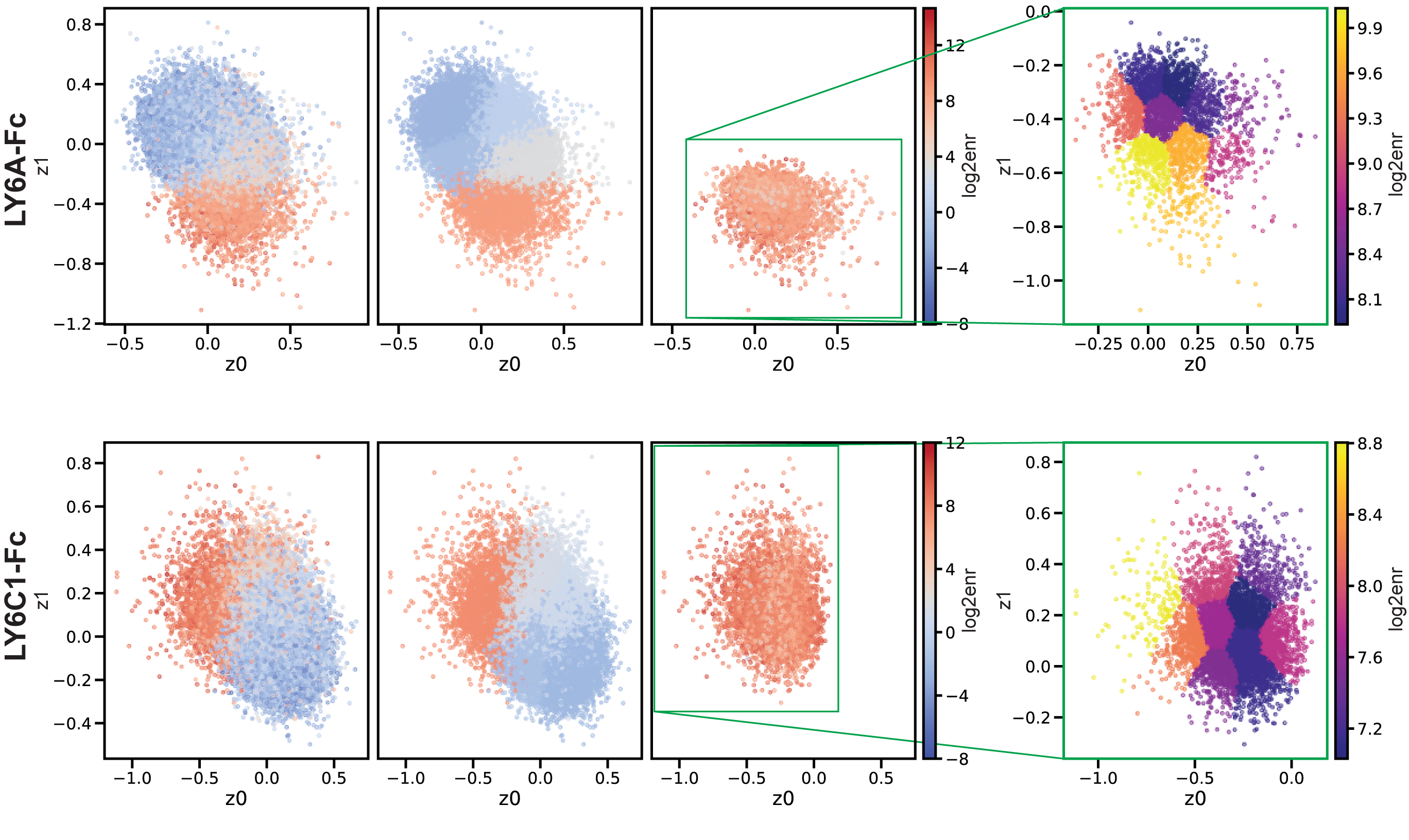

Supplement: S14 Fig — From left to right: all training points colored by assay log2 enrichment; all training points colored by mean primary cluster (see Materials and methods, SVAE Sequence Generation) log2 enrichment; top (highest mean enrichment) cluster colored by assay log2 enrichment; top cluster further clustered into subclusters, colored by mean subcluster log2 enrichment. The first 3 plots from the left share spatial axes and color scale; the rightmost subclustering plot is centered on its own axes and recolored on its own scale. The underlying data supporting S14 Fig can be found at https://doi.org/10.5281/zenodo.7689794: LY6A_SVAE_training_predictions.csv and at https://doi.org/10.5281/zenodo.7689794 LY6C1_SVAE_training_predictions.csv. (TIF) [file pbio.3002112.s014.tif]

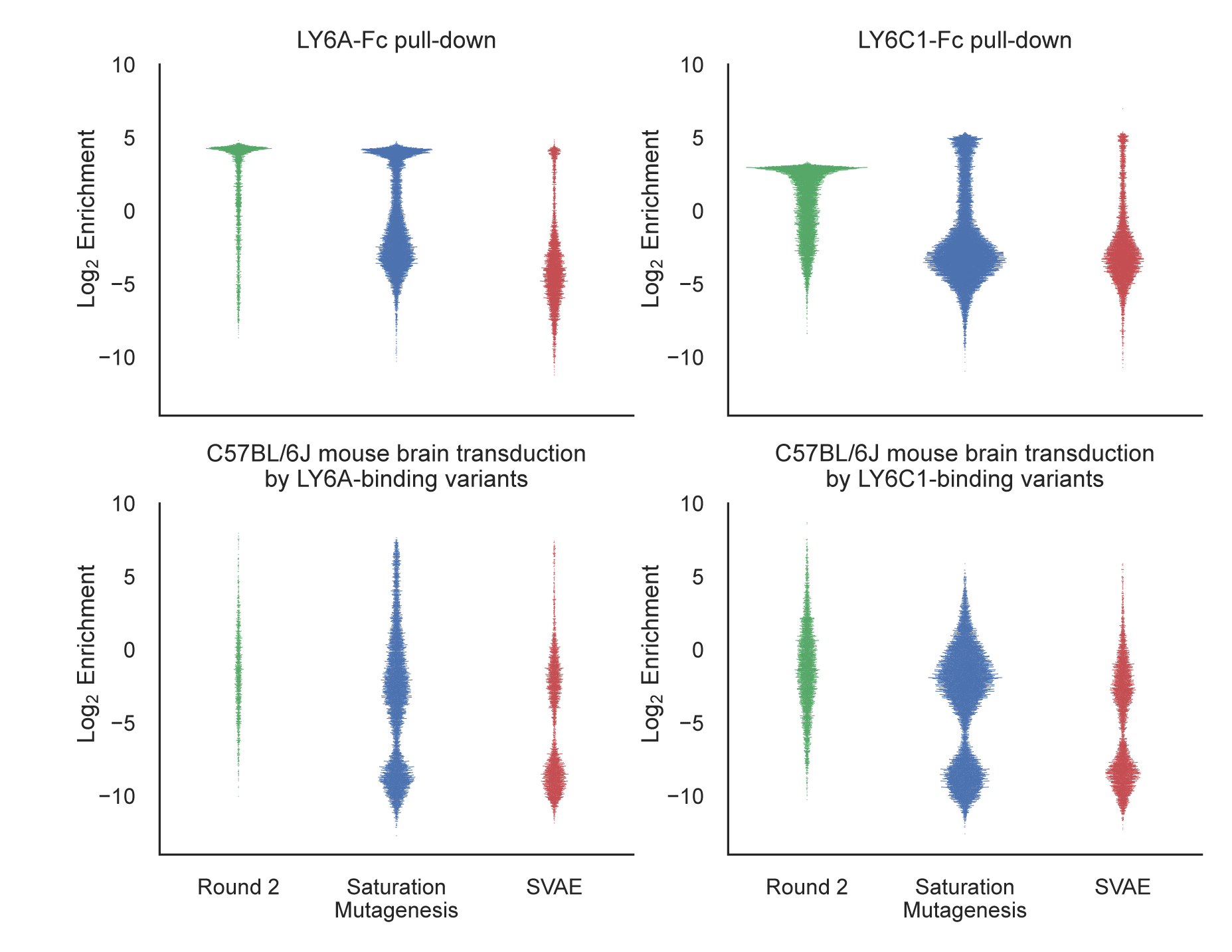

Supplement: S15 Fig — The data from Fig 4F (S12–S23 Data) is shown without clustering. The panels show the performance of variants for each assay: LY6A-Fc pull-down, LY6C1-Fc pull-down, C57BL/6J mouse brain transduction by LY6A-binding variants, or C57BL/6J mouse brain transduction by LY6C1-binding variants. As in Fig 4F, variants with a fitness value below log2 enrichment of −1.0 are excluded. The underlying data supporting S15 Fig can be found at https://doi.org/10.5281/zenodo.7689794: round2_codons_merged.csv and at https://doi.org/10.5281/zenodo.7689794: SVAE_SM_library_codons_merged.csv. (TIF) [file pbio.3002112.s015.tif]
