# Supplementary material for: Targeting AAV vectors to the central nervous system by engineering capsid–receptor interactions that enable crossing of the blood–brain barrier
Source: PLoS Biol. 2023 Jul 19;21(7):e3002112. doi: 10.1371/journal.pbio.3002112 (PMC10355383; doi:10.1371/journal.pbio.3002112)
Supplement: Attachment — Submitted filename: Response to reviewers.pdf [file pbio.3002112.s039.pdf]

Response to reviewers

We thank the editor and reviewers for their positive and constructive comments. We have addressed the data-related and editorial requests (A-G) as follows:

(A) We would like to suggest that the following modification the title:
"Targeting AAV vectors to the central nervous system by engineering capsid-receptor interactions that enable crossing of the blood-brain barrier"

We have adopted the editorial suggestion to change our title to "Targeting AAV vectors to the central nervous system by engineering capsid-receptor interactions that enable crossing of the blood-brain barrier".

(B) In the animal ethics statement in the Methods section, please provide the method of euthanasia used to sacrifice the mice

We have added the following to the Animals section in our Methods: "Mice treated at the Broad Institute were euthanized with a lethal dose of Euthasol (Virbac) or by cervical dislocation under the influence of isoflurane as approved under our IACUC protocol. Mice at Baylor were euthanized by cervical dislocation under the influence of isoflurane anesthesia, according to the approved Baylor IACUC protocol."

(C) You may be aware of the PLOS Data Policy, which requires that all data be made available without restriction: http://journals.plos.org/plosbiology/s/data-availability. For more information, please also see this editorial: http://dx.doi.org/10.1371/journal.pbio.1001797
Note that we do not require all raw data. Rather, we ask that all individual quantitative observations that underlie the data summarized in the figures and results of your paper be made available in one of the following forms:
-Supplementary files (e.g., excel). Please ensure that all data files are uploaded as 'Supporting Information' and are invariably referred to (in the manuscript, figure legends, and the Description field when uploading your files) using the following format verbatim: S1 Data, S2 Data, etc. Multiple panels of a single or even several figures can be included as

multiple sheets in one excel file that is saved using exactly the following convention: S1_Data.xlsx (using an underscore).

We have renamed all supplementary files to S# Data. Due to the large sizes of our datasets (>1M rows exceed Excel's maximum), it is not possible to put all of these data into Excel file(s).

- Deposition in a publicly available repository. Please also provide the accession code or a reviewer link so that we may view your data before publication.
Regardless of the method selected, please ensure that you provide the individual numerical values that underlie the summary data displayed in the following figure panels as they are essential for readers to assess your analysis and to reproduce it:
Figure 1B-E, 1H-I, 2B, 2D-F, 3C, 4D, S1A-B, S2A-J, S3C-D, S4A-B, S5A-I, S6, S7A-C, S8C, S9A-D, S12, S13
NOTE: the numerical data provided should include all replicates AND the way in which the plotted mean and errors were derived (it should not present only the mean/average values).

The library data files required to reproduce all plotted figure panels are too large (298 MB) to include as supplementary data and are therefore deposited on Zenodo at https://doi.org/10.5281/zenodo.7689794. The Supplementary Data files S1-S23 contain analyzed and processed values derived from the data available on Zenodo.

Our Data Availability statement now reads:

> All code used in this study is available on GitHub: https://github.com/vector-engineering/AAV_capsid_receptor/. All data required to reproduce each plot are available from the Zenodo open repository at https://doi.org/10.5281/zenodo.7689794. These data include RPMs and enrichment values from the libraries used in this study. Supplementary Data files S1-S23 contain analyzed and processed values derived from the data available on Zenodo.

In addition, we provide a comprehensive table pointing reviewers and readers to the supplementary data file(s) required to produce each plot.

*(D) Please also ensure that each of the relevant figure legends in your manuscript include information on *WHERE THE UNDERLYING DATA CAN BE FOUND*, and ensure your supplemental data file/s has a legend.*

As requested, we have added information on where the underlying data can be found at the end of each relevant figure legend.

(E) Please ensure that your Data Statement in the submission system accurately describes where your data can be found and is in final format, as it will be published as written there.

We have updated our Data Statement as described above.

(F) Please note that per journal policy, we do not allow the mention of "data not shown" or other references to data that is not publicly available or contained within this manuscript (line 399). Please either remove mention of these data or provide figures presenting the results and the data underlying the figure(s).

We have now removed the sentence in our Methods section originally at line 399: “In most cases, the beads were saturated with Fc-fusion protein as confirmed by the protein gel (data not shown).” This change does not affect the reader’s ability to replicate our work.

(G) Please also provide a blurb which (if accepted) will be included in our weekly and monthly Electronic Table of Contents, sent out to readers of PLOS Biology, and may be used to promote your article in social media. The blurb should be about 30-40 words long and is subject to editorial changes. It should, without exaggeration, entice people to read your manuscript. It should not be redundant with the title and should not contain acronyms or abbreviations. For examples, view our author guidelines: https://journals.plos.org/plosbiology/s/revising-your-manuscript#loc-blurb

We provide the following blurb for our publication:

Directly targeting adeno-associated virus capsids to specific proteins expressed on the blood-brain barrier can rapidly generate potent gene delivery vectors with predictable *in vivo* tropisms.

REVIEWS:

Reviewer #1 (Thomas J McCown, signs review): The manuscript "Targeting AAV vectors to the CNS via de novo engineered capsid-receptor interaction" presents studies that characterize a novel means to engineer novel AAV capsids through in vitro receptor binding. The overall approach arises from a sound rationale and the use of crystallizable fragment domain binding (FC fusions). Given that the receptors for AAV blood brain transit in mice have been identified, these two receptor proteins, LY6A and LY6c were constructed as FC fusions. Then AAV libraries of 7 mer insertions between 588 and 589 of AAV9 VP1 were interacted with the FC fusions and subsequently pull down assays yielded novel capsids that bound either of the two receptor protein. Substantial subsequent studies characterized these novel capsids in comparison to reference capsids such as AAVF and PHPe.B. Extensive in vitro and in vivo studies validated the technique as a means to generate novel AAV capsids that
cross the mouse BBB and transduce cells in the mouse brain. As such the findings will be of interest to the AAV gene therapy community. Certainly the findings present a novel means to generate new AAV capsids across species. The only concern involves a method of in vivo validation. In several instances, mRNA determinations were used to indicate in vivo transduction. However, a direct correlation between mRNA and the in vivo amount of viral particles present, as well as the level of protein expression remains tenuous. A tangential example of this arises in figure 3C-D. The enrichment of BI-28 and BI-65 appear virtually identical, but in the individual in vivo validation, GFP expression is vastly different between the two viruses where BI-65 exhibits much lower overall expression compared to BI-28. This divergence should provide an element of caution to the extensive discussion of machine learning applications. Although ML has substantial merit, the final outcome depends upon the content of the learning trials. Also, the general discussion of applicability to other paths of AAV biology should mention that to utilize this technique, it will be important to identify what part of VP1, VP2 and/or VP3 mediate the element to be optimized. In summary, the present studies establish a novel means to generate chimeric AAV capsids that may have enhanced properties and should prove interesting to a wide gene therapy audience.

We thank Reviewer #1 for their assessment of our work as novel, rational, and of interest to the gene therapy community. We agree that *in vivo* validation remains a major challenge in AAV engineering. The readouts of AAV library screens typically consist of either DNA or mRNA-based enrichment scores. These values from *in vivo* studies provide a metric by which to rank the function of sequences relative to each other. While these data generally accurately predict the relative *in vivo* function when capsids are tested individually, the library data does not always perfectly match the ranking observed when tested individually. There are multiple hypothetical reasons that could account for these minor discrepancies. First, there is bias introduced by production fitness, e.g., variants that are rare in the starting library may have inflated enrichment scores. This bias is not present when testing viruses individually. Second, the amount of capsid mRNA produced from individual transduced cells will vary, especially in an organ like the brain where there is a great diversity of cell types. Despite these potential biases, we show here that the library data can be effectively used to (1) pick individual top hits from the selection and (2) train generative ML models.

We also agree that the outcomes of ML depend on the nature of the training data and that ML should be applied to answer questions that can be feasibly addressed given the type of training data available. In our approach, we use ML to predict new capsids that interact with a target receptor, i.e., our objective is to use the ML as a method for affinity maturation/optimization by expanding the sequence space explored in a second-round library. The value of ML in this application is in optimizing the search for more functional sequences so that it is cost-effective and practical (as compared to performing saturation mutagenesis on every motif identified in first round screening). The training data produced by our receptor-targeted approach is well suited for this purpose without having to precisely score the *in vivo* performance of variants relative to each other. However, we have a separate manuscript available, Eid et al. 2022 *bioRxiv*, that addresses the question of how training data and models may be generated that can more accurately rank variants against each other.

All of our screens were performed such that the engineered 7-mers were inserted into all capsids subunits (VP1, VP2, and VP3). Evaluating these novel 7-mers as mosaics with wild

type or other engineered capsids is an interesting application that is beyond the scope of this study.

Minor -Should define ML when it first appears on line 71

Thank you for catching this. We have now defined ML where it is first mentioned in our introduction.

Reviewer #2 (Casey A. Maguire, signs review): The manuscript Targeting AAV vectors to the CNS via de novo engineered capsid-receptor interactions by Huang et al is a proof-of-concept study with the intent of driving selection of efficient AAV vectors for in vivo transduction using in vitro binding assays directed towards specific ligands.
The approach is reminiscent of the early days of phage biopanning to isolate peptides binding to target receptors, which used phage libraries binding to microtiter wells coated with extracellular domains of receptors, except this approach is done using the peptide display library in the context of AAV capsids. The authors purified recombinant Ly6a-Fc and Ly6c-Fc and conjugated these proteins to beads and incubated an AAV9 peptide display library to identify capsids which could bind to either receptor. Using clustering techniques on the NGS data,they then identified motifs that were associated with either Ly6a or Ly6c binding. Finally, they picked a subset of these candidates for in vivo testing in Ly6a expressing mice (C57BL/6) or Ly6c expressing (C57BL/6 and BALB/c) and showed that, as predicted Ly6a binding peptides only transduced C57 and the Ly6c binding peptides transduced both strains of mice, aligning with the receptor expression profile of these mouse strains.
There are two exciting findings that came about with this work. First, it appears that the Ly6 family of receptors on the BBB of mice is an efficient conduit for AAV-mediated BBB penetration and transduction. This was partially known from the prior studies with PHP.B and Ly6a, but greatly extended by this work to Ly6c and other capsids. The authors appear to unravel the mystery of the BBB-penetration mechanism of many of published engineered capsids from the Gradinaru, Deverman, Maguire, and Nonnenmacher groups, which is very interesting and important to know (particularly in their lack of potential to translate to human for BBB penetration). Second, the use of specific ligands to direct tropism is used

successfully here to drive the desired phenotype: BBB penetration and brain transduction after systemic delivery.
Overall, the manuscript is well written, and the extensive experiments and extensive data analysis appear to be well done. This is a very interesting and thought-provoking approach, although it is currently unclear how it will turn out for target ligands that are currently not known to allow the complete transduction process of AAV. However, as more knowledge comes in on the vector biology front, it may be put to good use.

Comments below are suggested to improve the study.
Major Comments.
Comment 1. While the results using Ly6a and Ly6c binding to drive selection of efficient capsids was demonstrated here to work very well at identifying capsids that crossed BBB and transduced the brain, the authors had a priori knowledge that Ly6a would satisfy the entire transduction process. As the authors know (and discuss somewhat), binding to a receptor is only one part of the transduction process and identifying other "AAV-transduction" permissive receptor targets may not be as straightforward. For many years, in vitro selections with AAV vectors have been performed with very little success at translating to desired in vivo transduction properties.
There seems to be a big "what now?" ending to the paper. It would be really useful to understand the authors thinking on how this strategy will be employed in the real world to identify clinically relevant AAV vectors.
Could the authors speculate on some potential targets or how to enhance this methodology to increase the chances of targeting receptors that would mediate functional transduction?
Also the statement "This work demonstrates that AAV capsids can be directly targeted to specific proteins to generate potent gene delivery vectors with known mechanisms of action and predictable tropisms" should be tempered somewhat in light of the factor that there was a priori knowledge that the chosen receptor family mediates efficient in vivo transduction in mice. It is currently unknown how this will translate to new receptors that we know exist, just not in the context of AAV transduction permissivity.

We thank Reviewer #2 for their appreciation of our exciting findings and their insight that identifying target proteins that can mediate AAV transduction is the challenging and invigorating next step of extending our approach. Using the same approach described in

this manuscript, we have developed capsids that engage human proteins present on the CNS endothelium, but testing these capsids *in vivo* is slowed by the need to generate mice that express the human form of the target receptor gene. We are in the process of characterizing these capsids and believe there are likely dozens of promising human receptors to target gene delivery vectors to disease-relevant cell types. In support of this, researchers in the Gradinaru laboratory at Caltech (*Shay et al. bioRxiv, 2023*) have also determined that two previously described capsids 9P31 and 9P36 (*Nonnenmacher et al. MTMCD, 2020*) can use the mouse carbonic anhydrase IV (Car4, CA4 in humans) protein to cross the BBB. These are the same two previously published capsids that we did not identify as LY6A or LY6C1 binding capsids (Fig 2F). The identification of mouse Car4 as a receptor for an engineered AAV is exciting as it extends the types of proteins that can be considered as targets beyond the Ly6 family. Interestingly, Car4/CA4 shares several features with Ly6a and Ly6c1, notably that they are all GPI-anchored membrane proteins that are highly expressed on brain endothelium. Encouragingly, unlike Ly6a and Ly6c1, carbonic anhydrase IV (CA4) is present in rodents and primates. Although the primary sequences of Car4/CA4 diverge significantly across species, and 9P31 and 9P36 do not appear to bind primate forms of the CA4 protein, it may be possible to engineer AAVs that cross the BBB by engaging the human CA4 protein.

We have now added new text about these recent developments in our discussion: "Recently, Shay *et al.* have identified Carbonic Anhydrase IV [43] as the cellular protein co-opted by the two previously reported mouse BBB-crossing AAVs (9P31 and 9P36) that we reported here do not engage LY6A or LY6C1 [3]. Like LY6A and LY6C1, Carbonic Anhydrase IV is a GPI-anchored protein that is highly expressed on CNS endothelial cells. Notably, this protein is present in both rodents and primates, and may therefore represent a prime target for engineering receptor-targeted AAVs with a predictable mechanism of action for human CNS gene therapy."

Comment 2. What were the cellular targets in the brain of the identified capsids (BI48, BI49, BI28, BI62, BI65)? Were any "detargeted" from liver?

Capsids that cross the BBB using LY6A or LY6C1 to enter the mouse CNS, seem to share the ability to transduce neurons, astrocytes, oligodendrocytes, and endothelial cells,

although some capsids show partial bias toward specific cell types like endothelial cells and astrocytes (e.g., AAV-PHP.V1). As fewer potent LY6C1 binding capsids have been reported and characterized we chose our overall best performing LY6C1 binding capsid BI28 for further analysis. We now show new data in S9A-C Fig that AAV-BI28 transduces neurons and glia as assessed by colocalization with NeuN, S100, and APC (CC1) positive cells. AAV-BI28, and LY6C1 binding capsids more generally, are highly effective at transducing astrocytes. Therefore, we also assessed the ability of AAV-BI28 to mediate gene editing in astrocytes throughout the adult brain. For this experiment, we used AAV-BI28 to deliver a dual vector SaCas9-based editing system (AAV-GfABC1D-SaCas9 and AAV-GfABC1D-NLS-GFP-gRNAs) designed to turn on expression of the tdTomato reporter in Ai9 mice (S9D-I Fig). We observed tdTomato expression in a significant fraction of astrocytes throughout the CNS of mice that received both vectors. As part of the NIH Somatic Cell Genome Editing Program, we sent the AAV-BI28 vectors for independent validation. The independent team at Baylor College of Medicine was able to reproduce our editing results in a separate cohort of mice. These new data and methods are briefly described in the main text and the results are shown in the new S9 Fig.

Based on library expression data from the liver, none of the individually validated capsids described in this study are more detargeted from the liver than previously described variants (AAV-PHP.eB and AAVF). The liver enrichment scores for each variant are now included in the new S8C Fig.

Comment 3. Could the authors speculate why the Ly6 family of receptors are such promoters of efficient AAV BBB penetration and transduction (at least in mice)?

We had speculated in our 2019 publication describing the LY6A-dependent mechanism of AAV-PHP.eB that some favorable characteristics of receptors that could mediate efficient AAV crossing of the BBB might include (1) abundant luminal surface exposure on brain endothelium, (2) localization within lipid micro-domains through GPI anchoring, or (3) specific recycling/intracellular trafficking capabilities. Further support for the use of highly expressed GPI anchored proteins for mediating efficient BBB crossing of engineered AAVs comes from a recent preprint (Shay et al. bioRxiv 2023) that identified mouse Carbonic Anhydrase IV as a receptor for the previously described 9P31 and 9P36 capsids

(Nonnenmacher et al. 2021). However, natural AAVs receptor interactions are not restricted to GPI-anchored proteins and include a diverse set of molecules including, AAVR, integrins, and a variety of glycans. We expect to glean additional information about what types of receptors to target through additional screening efforts.

Comment 4. While the data is highly suggestive of Ly6c pulled-down capsids in vitro mediating transduction using Ly6c receptor in vivo, this has not been definitively demonstrated by the presented data. Could the Ly6c (and for that matter Ly6a) receptor transduction dependence of the identified capsids be demonstrated at least in vitro with the Ly6c1 and Ly6a lines generated in the authors' 2019 PLOS paper?

We validated the LY6A and LY6C1 dependence of the enhanced binding and transduction phenotypes of these capsids *in vitro* as part of our library screen using exogenous receptor expression assays in HEK293 cells. These data are now included as part of the new S8 Fig.

Minor comments.
Comment 1. It was interesting to see the AAV-F peptide sequence (FVVGQSY) mediates Ly6c binding and many of these amino acids (5/7) were observed in some of the pull-down derived capsids (e.g. FVYGQIA, Fig S6) as well as the MDVIA capsid from the Sabeti group (5/7 amino acid identity). This further suggests that this motif is strongly associated with Ly6C binding and should be included in the text (results or discussion).

The *in vitro* pull down assays identified a large number of sequence motifs, including ones encompassing AAVF, that were capable of mediating interactions with LY6A or LY6C1 that led to dramatically enhanced *in vivo* CNS transduction. This particular motif was not observed in as many sequences as other motifs, which is why we chose not to highlight it in Fig 1 or with specific descriptions in the text. Nevertheless, our library enrichment scores and individual validation study independently validated the potent CNS transduction of the AAVF capsid.

Comment 2. Could the authors discuss how this strategy could be used to select for "copy-cat AAV's" in the future and the potential implications? For example if a new receptor is

identified by group A to mediate efficient AAV transduction of a particular capsid, group B could use this in vitro pull down technology to identify capsids with similar properties.

The approaches we describe here should make it possible for researchers with the appropriate expertise to identify unique capsids that bind the same target. This is no different than what occurs in the development of other biologics (e.g., antibodies or nanobodies) where numerous researchers and companies have developed antibodies that recognize a common target. Like with these other molecules, capsids that bind a single target can differ substantially in their functional characteristics beyond receptor binding, thus careful and thorough screening is an essential component of finding the capsids that perform best across critical functional metrics.

Reviewer #3: Huang et al. provide a detailed quantitative evaluation of AAV capsid variant selection based off of in vitro affinity based binding followed by in vivo validation in two strains of mice known to differentially express the receptor Ly6A. Importantly, the authors evaluate the ability of a SVAE Machine-learning model to generate an AAV capsid variant library enriched for production-competent and high target receptor binding capsids. The approach to select capsids based off of cell-free receptor binding is compelling and has the potential to yield enriched AAV variants that may be rationally pre-selected for transducing in tissues.

While it is attractive to think that novel AAV capsids with high target cell transduction can be generated with minimal cycling in animal models and at a rapid rate, one potential concern is that binding of specific protein targets may not translate into other steps critical for viral infection such as uptake/cell entry. This aspect should be highlighted as a potential limitation.

Another potential limitation is the species-specific difference often seen in such protein based targets (from a sequence perspective) and the implications for species-selectivity that needs to be discussed. Nevertheless, while the study does not provide any significant new insight into AAV biology (from using previously identified receptors in mice), the incorporation of the SVAE machine learning technique exemplifies the power of this technique in being able to predict infectivity and fitness from a vector production standpoint.

Based on comments from both Reviewers #2 and #3, we have now added wording to our discussion to highlight the challenge of identifying cellular proteins that can act as *de novo* AAV receptors.

The reviewer is correct that species-specific differences will remain a challenge with this *in vitro* approach except in cases where the target protein is highly conserved. We point out the encouraging findings recently reported by other groups who have identified proteins that exist in NHPs and humans with the ability to facilitate BBB-crossing of engineered AAVs. It is also important to mention that *in vivo* AAV selections have, with only a few exceptions, also yielded species-specific capsids. And given that the receptor target is not usually obvious, *in vivo* selections yield capsids with unknown translational potential.

This manuscript is likely the first of many, from our group and others, that will use *in vitro* receptor binding assays to rapidly identify gene delivery vectors with known mechanisms of action and predictable species or cross-species tropisms.

Overall, from a methods/resource contribution perspective, the authors provide a detailed validation of this approach, adequate controls (e.g., comparing and contrasting sequences enriched for Ly6A and Ly6C1 binding compared to Fc binding alone) and the ability of the machine learning approach combined with in-life selection as being on par with in life selection alone.

Additional comments:
In figure 4F, the recovered enriched sequences are plotted according to cluster log size and cluster max enrichment. Since the data are available and easily sortable, e.g., in Figure S13, the authors should consider plotting enrichment of brain transduction for individual sequences within the SVAE and Sat. mut. Libraries, either in a new Figure 5, or as a subpanel of figure F? This would enable further visual understanding of the performance of the SVAE library in generating individual top enriched amino acid sequences.

We have updated our S13 Fig (this is now S15 due to the addition of two new supplementary figures) to plot the *in vitro* binding and brain transduction enrichment scores of individual Round 2, saturation mutagenesis, and SVAE variants. The panels show the

performance of individual variants for each assay: LY6A-Fc pull-down, LY6C1-Fc pull-down, C57BL/6J mouse brain transduction by LY6A-binding variants, or C57BL/6J mouse brain transduction by LY6C1-binding variants. The plots show that both approaches generated top performers in *in vitro* target binding and *in vivo* brain transduction compared to the top hits from the Round 1 screen.

Figure S13 as referenced in the text contains the enrichment scores for only the LY6C1 library, while figure 4F clearly plots both the LY6C1 and LY6A libraries. As it stands, there is no textual reference to the quantitative data supporting the LY6A plots in figure 4F. The authors should consider including in the text the reference to the appropriate table listing the LY6A enrichment related to Figure 4F?

S13 Fig (now S15 Fig) shows plots of the enrichment scores for both LY6A and LY6C1-binding variants. The table listing the LY6A enrichment related to Figure 4F and S15 Fig are: S12, S16-20 Data; for LY6C1, these are: S13-15, S21-23. We have now cited these data collectively (S12–23 Data) in the caption for Fig 4F and S15 Fig.
